# Adolescent health in relation to their peers: likeability as a resilience factor

**DOI:** 10.1016/j.ssmph.2025.101833

**Published:** 2025-06-25

**Authors:** Jaap Nieuwenhuis, Daniël Veennema

**Affiliations:** a Department of Sociology, University of Groningen, the Netherlands; b Faculty of Behavioural and Social Sciences, University of Groningen, the Netherlands

**Keywords:** Social networks, Health, Peer influence, Likeability, Taiwan

## Abstract

Adolescence is a period in which health-related behaviors develop. Peer group influence plays an important role in this development. Some adolescents are, however, more or less resilient to peer influence. We argue that students’ likeability amongst their classroom peers functions as a resilience factor in the relation between the health of classroom peers and their own health. We test this by studying 7th grade students from the Taiwan Youth Project (N = 2350) in 81 classrooms, examining the differences in individual and classroom health one year later. The results show that there is indeed a contextual effect of classroom health on individual health, but this effect is weaker to nonexistent, the better liked a student is. Students who score low on likeability are most susceptible to peer influence. The moderation of likeability is only found for boys. Our study shows that likeability is an important resilience factor to social influence and offers insight in how contexts differentially shape individual behaviors.

## Highlights

• The health of classroom peers is positively related to individual health.
• Likeability functions as a resilience factor against peer influence.
• Adolescents who are better liked are not susceptible to the influence of classroom peers on their health.
• The moderation of likeability is only found for boys.

## 1 Introduction

Adolescence is a period in people's lives where they become more independent from their parents and start developing their own opinions and habits. The relative decline of parental influence is substituted by the relative increase of peer influence. Young adolescents model their behavior after peers (Sumter et al., 2009), making the study of peer influence relevant for the development of most behaviors, including health-related behaviors, such as smoking and drinking. During adolescence, many people will start engaging with these health behaviors for the first time, which will influence how behavior spreads throughout the group.

However, we also know that peer groups are not absolute homogeneous cliques. Rather, they contain adolescents who behave more like the majority of the group and others who do not follow the dominant patterns of behavior. This suggests that some adolescents are more likely to be influenced by peers than others. To better understand the underlying mechanisms, it is crucial to study peer influence in the light of relational characteristics, specifically, characteristics that make people susceptible or resilient to peer influence. Because peer influence is a network process, it is important to understand the position of adolescents within these networks. One characteristic that is argued to be a buffer against peer influence is popularity. The prioritization of popularity is most prominent in early adolescence (Sentse et al., 2014). Adolescents who are popular are so, because of certain behaviors such as assertiveness, risk-taking, and dominance. Popular adolescents set the norms in a group and exert their influence over the group in several ways (Laursen & Veenstra, 2021). One problem with using popularity as a moderator of peer influence is that the role of popularity is ambiguous. Popular adolescents may be more susceptible to peer influence as they seek to maintain their social standing, while at the same time, nonpopular adolescents may also be more susceptible to peer influence in order to gain more social standing. This contradiction suggests popularity is not a good measure for resilience (Allen et al., 2005). We argue that, instead of using popularity to examine whether adolescents are susceptible to peer influence, it is better to use likeability. The concept of likeability deals with more genuine relationships. Adolescents who are liked generally engage in more prosocial behaviors, such as kindness, empathy, and cooperation (Cillessen and Lansu, 2011). Popular adolescents can be, but are not necessarily well-liked adolescents (Cillessen and Rose, 2005). Because, unlike popularity, likeability is not derived from engaging in certain normative (health-related) behaviors, it is much more suitable to study as a potential buffer against peer influence on health behaviors.

To study peer effects on health in classrooms, we focus in on Taiwan using data from the Taiwan Youth Project. Taiwan is a suitable context to study this phenomenon, because in Taiwanese middle school (7th to 9th grades), students typically spent a long amount of time each day for three years with the same group of peers (a school day is about 10 h). Because teachers rotate between classrooms and students have their own designated classroom, peers are bound to a specific place. This gives ample time and opportunity to form social networks. This context is very apt to study adolescents' peer networks, because we likely capture a larger proportion of them and with that come closer to potential mechanisms. However, it also warrants some rethinking when translating the results to other contexts. Within this context, we ask the question: How does likeability moderate the relation between students’ health and that of their classroom peers?

### 1.1 Peer effects

The move from primary to secondary school is an exciting time for many adolescents, and can bring on many challenges, such as how to deal with their peers (Lucey & Reay, 2000). Adolescents find themselves in a classroom with peers with whom they are in close contact and spent a large part of their formative years with. This space is where adolescents get exposed to many new beliefs and behaviors. Among these new behaviors many have an impact on their health. Around this time, people may start smoking and consuming alcohol for the first time, they get new hobbies, and change the way they eat (Fortin & Yazbeck, 2015). To help navigate this difficult time, adolescent might compare their own behavior to that of their peers to seek approval or acceptance (Arango-Paternina et al., 2022; Fortin & Yazbeck, 2015). New friendships help give stability in this new environment. In these friendships, similarity in behaviors and beliefs can help maintain and create relationships. The changing health behaviors of a group of adolescents will influence the group overtime. As new behaviors become the norm, people will feel compelled to conform to these norms to fit-in with the larger group (Arango-Paternina et al., 2022). If you do not conform to these norms, you can come to be seen as an outsider who does not respect the social rules of the classroom. Adolescents that do not change their behavior thus risk being socially outcast (Cruz et al., 2012). If the health-related behaviors in a classroom indeed converge, the health of students in the same classroom will also converge. Therefore, we expect that the average classroom health will be positively related to individual students’ health in that classroom.

The role of peers in health behavior has been studied extensively, for example, adolescents are more likely to drink alcohol or smoke when their peers do (Cruz et al., 2012; Green et al., 2013). Peers' mental health, weight management, and physical activity have also been positively related to own behaviors (Conway et al., 2011; Marks et al., 2015; Rancourt & Prinstein, 2010). The most evidence available seems to be present for the link between group physical activity and own physical activity (Macdonald-Wallis et al., 2012; Prochnow et al., 2020). However, the literature also has many mixed and null findings (Crudgington et al., 2023; Letina et al., 2024; Montgomery et al., 2020), suggesting that there is no clear-cut effect of peers’ health behaviors. It is likely that the heterogeneity within research samples leads to this diversity in conclusions, and that we need to look more closely at the characteristics of adolescents who are and who are not influenced by their peers.

### 1.2 Likeability as resilience

Peer influence appears to be a conditional mechanism. Not every behavior will be adopted by the broader classroom if a few adolescents start doing it. In a classroom full of smokers, some adolescents will still not pick up smoking themselves, while others may start doing it, because their peers are doing it (Fortin & Yazbeck, 2015). The theory of differential susceptibility (Belsky & Pluess, 2009) helps explain this difference in influenceability. Some adolescents will be more resilient than others, who might be more susceptible to peer influence. Being more resilient translates into having more security or confidence in your own behaviors and attitudes. This security means not feeling pressured to change when peers do or to conform to group norms about behavior. More resilient adolescents are more likely to make their own decisions, which still means they might start smoking, it will however be unrelated to the smoking behavior of their peers. Within a group context like a classroom, it stands to reason that one's social position within that group may contribute towards their level of resilience. Resilience is not an indicator of health, but resilience describes how a person handles external influences that might have an influence on their health.

As an indicator of someone's social position within a group, likeability, also referred to as social preference, plays an important role in the life of many adolescents (Lansford et al., 2007). Particularly the lack thereof can play a detrimental role, as being deemed unlikeable is a form of peer rejection. Peer rejection has a negative impact on the well-being of students. They might experience mental issues due to their rejected status (Lansford et al., 2007), or they might turn towards unhealthy behaviors such as smoking as a coping mechanism (Aloise-Young & Kaeppner, 2005Aloise‐Young & Kaeppner, 2005). Likeability has not been widely researched in the context of health. Several studies investigated if likeability is related to substance use, mental health and disordered eating, but the results are inconclusive (Cole et al., 2024; Ennett et al., 2008; Smink et al., 2018; Yang et al., 2020). By studying likeability in combination with peer effects, we will be able to look into differential effects of likeability and potentially shed more light its role for health.

Besides having a direct impact on health, likeability is likely to be a source of resilience to peer influence. Students who are better liked by their peers, may not need to succumb to peer pressures, as they do not seek further recognition, while students who are less liked, may still want to conform to behavioral norms in order to fit in, in an attempt to boost their likeability. Thus, adolescents unhappy with their likeability status will see certain common behaviors in the classroom, and try to emulate these behaviors. For example, the less liked adolescents might turn to (excessive) drinking in an attempt to improve their social standing (Choukas-Bradley et al., 2015). In similar findings, it was shown that adolescents with low likeability or social status were more susceptible to start smoking when their peers did compared to their better liked or higher status counterparts (Ennett et al., 2008; Otten et al., 2008). We can understand likeability as a buffering factor. Likeable students will feel less pressured by the norms of the classroom, and will thus also be relatively less affected by the general health level in the class. For the adolescents who are less liked, there is much more to win or lose by conforming to the norms of peers in the classroom, and therefore they are more likely drift towards the classroom average. Because peer influence tends to differ between boys and girls (McCoy et al., 2019), we additionally examine sex differences.

## 2 Data and methods

### 2.1 Data

In this study we use data from the Taiwan Youth Project (TYP), a longitudinal panel that ran since 2000 on students and parents from northern Taiwan. The original sample consisted of 5541 students, divided over two cohorts: a 7th grade cohort, who were in the 1st year of middle school (51.5 %) and a 9th grade cohort, in the 3rd year of middle school (48.5 %). We use only data from the 7th grade cohort for this study. Students were sampled from three regions (Taipei city, Taipei county, and Yilan county), 40 schools, and 162 classrooms. For this study we use the survey waves from the 7th and 8th grade, when students were, on average, 13 and 14 years old, respectively. Most measures were taken from the 7th grade wave, except students’ health, which was also taken from the 8th grade wave. After listwise deletion of cases with missing values the sample size is 2350 adolescents, clustered within 81 classrooms.

### 2.2 Measurements

Dependent variable health status is measured twice, once in the 7th and once in the 8th grade, with the question: “How is your current health condition?” Answering categories were, 1) very poor, 2) poor, 3) fair, 4) very good, and 5) excellent. The development of health status between the 7th and 8th grade were measured by subtracting the answers from the 7th grade from the answers from the 8th grade. The descriptive statistics of these and other variables are available in Table 1.

**Table 1:** Descriptive statistics (N = 2350).

Variable	Mean/Prop.	S.E.	Min.	Max.
Health status 8th grade	2.76	.84	0	4
Health status Δ7^th^-8th grade	−.00	.94	−3	4
Average classroom health status 7th grade	2.75	.19	2.22	3.29
Average classroom health status Δ7^th^-8th grade	.01	.20	−.49	.47
Reciprocated relationships	1.37	1.21	0	5
Non-reciprocated outdegree	1.49	1.36	0	5
Non-reciprocated indegree	1.53	1.65	0	12
School average income	58.37	12.61	34	87.95
Household income (log)	3.89	.69	.69	5.05
Parents' education	3.42	1.60	1	7
Female	.49			
Location: Taipei city	.40			
- Taipei county	.39			
- Yilan county	.22			
Ethnicity: Minnan	.77			
- Hakka	.08			
- Mainland	.13			
- Aboriginal or other	.03			

Average classroom health status is measured twice, by taking the mean of the health status variable above of all respondents who are in the same class, once in the 7th and once in the 8th grade. To measure the change between the two years, the 7th grade variable is subtracted from the 8th grade variable.

To calculate the social network variables, students were asked to select up to five of their closest friends. Because the data collection targeted full classes in schools, many of these friends were identifiable in the data. This information was used to calculate outgoing (outdegree) and incoming friendship selections (indegree). *Outdegree* is the number of selected friends, ranging from 0 to 5. *Indegree* is the number of times a student was selected by others as a friend, which ranges from 0 to 14. *Reciprocated ties* were measured as all matching indegree and outdegree selections, meaning that only if student X selected student Y *and* student Y selected student X, they will have a reciprocated tie. This can range from 0 to 5. The variables used in the analyses are *non-reciprocated indegree* and *non-reciprocated outdegree*, which were calculated by subtracting reciprocated ties from indegree and outdegree, respectively. This was done to avoid having the same relationships counted more than once in the same analyses.

Household income was reported by parents in absolute numbers, and substituted with student-reported household income when the parent-reported measure was missing. The variable was measured in units of NT$1000 and was logged. School average income takes the average of the non-logged household income for all respondents who go to the same school.

Parents' highest level of education was measured as: 1 = elementary school, 2 = middle school, 3 = vocational high school, 4 = academic high school, 5 = junior college, 6 = university, and 7 = graduate school. Gender was coded: 0 = male, 1 = female. Location had three categories: 1 = Taipei city, 2 = Taipei county, 3 = Yilan county. Ethnicity was measured as the father's ethnicity: 1 = Minnan, 2 = Hakka, 3 = Mainlander, 4 = Aboriginal/Other. This was substituted with the mother's ethnicity when the father's ethnicity was missing.

### 2.3 Analytical strategy

To estimate the effect of contextual school class health and social networks on students' health status, we ran two sets of two regression models. The first set uses school class measures at the age of 13 (7th grade) to estimate students' health at the age of 14 (8th grade). The second set tests whether the difference in average health in the school class between the 7th and 8th grade predict the difference in students' health between the 7th and 8th grade. For both sets of analyses, the first model includes all covariates and the second model includes the interaction effect between classroom health (change) and indegree, to test whether likeability moderates the relation between classroom health (change) and students' health. From these interactions, marginal effects of the association between indegree and classroom health status and adolescents’ health status will be calculated for different values of classroom health status and indegree, respectively. This will give insight in how the moderation effect looks like. Because students are nested in classrooms, standard errors are clustered in classrooms. Finally, the differences between boys and girls were explored through a three-way interaction between gender, indegree, and change in classroom health.

## 3 Results

The results in Table 2 show that the average classroom health status is indeed related to individual students' health status. First, model 1a shows that the average classroom health in the 7th grade is predictive of students' own health in the 8th grade. And second, model 2a shows that change in the average classroom health status between the 7th and 8th grade is positively related to change in students’ own health status between those two grades.

**Table 2:** Regression results (N = 2350).

	Model 1: Health status 8th grade				Model 2: Health status Δ7^th^-8th grade			
	Model 1a		Model 1b		Model 2a		Model 2b	
	B (SE)	p	B (SE)	p	B (SE)	P	B (SE)	p
Average classroom health status 7th grade	.20 (.08)	.016	.06 (.10)	.590				
Average classroom health status Δ7^th^-8th grade					.98 (.03)	<.001	1.22 (.08)	<.001
Reciprocated relationships	.02 (.01)	.134	.02 (.01)	.123	−.03 (.02)	.053	−.03 (.02)	.062
Non-reciprocated outdegree	.02 (.01)	.078	.03 (.01)	.070	.01 (.02)	.525	.01 (.02)	.476
Non-reciprocated indegree	.01 (.01)	.508	−.27 (.11)	.020	−.01 (.01)	.434	−.01 (.01)	.497
Interaction classroom health status & indegree			.10 (.04)	.017			−.16 (.05)	.001
School average income	.00 (.00)	.005	.00 (.00)	.004	.00 (.00)	.089	.00 (.00)	.055
Household income (log)	.02 (.03)	.561	.01 (.03)	.596	−.04 (.03)	.130	−.05 (.03)	.109
Parents' education	−.02 (.01)	.066	−.03 (.01)	.062	−.01 (.01)	.344	−.01 (.01)	.336
Female	−.25 (.01)	<.001	−.25 (.04)	<.001	−.01 (.04)	.685	−.01 (.04)	.719
Location: Taipei city	(ref.)		(ref.)		(ref.)		(ref.)	
- Taipei county	.02 (.03)	.592	.02 (.04)	.563	−.02 (.02)	.250	−.02 (.02)	.345
- Yilan county	.08 (.05)	.085	.08 (.05)	.078	−.02 (.02)	.502	−.01 (.02)	.625
Ethnicity: Minnan	(ref.)		(ref.)		(ref.)		(ref.)	
- Hakka	−.01 (.06)	.819	−.01 (.06)	.832	.05 (.07)	.428	.06 (.07)	.391
- Mainland	.01 (.05)	.801	.01 (.05)	.833	−.05 (.06)	.403	−.06 (.06)	.354
- Aboriginal or other	−.26 (.11)	.023	−.26 (.11)	.025	.09 (.13)	.522	.08 (.13)	.528
Intercept	1.98 (.27)	<.001	2.38 (.32)	<.001	.14 (.10)	.174	.13 (.10)	.202
F	7.31	<.001	6.80	<.001	87.91	<.001	88.91	<.001
R^2^	.034		.035		.048		.051	

**Fig. 1:**
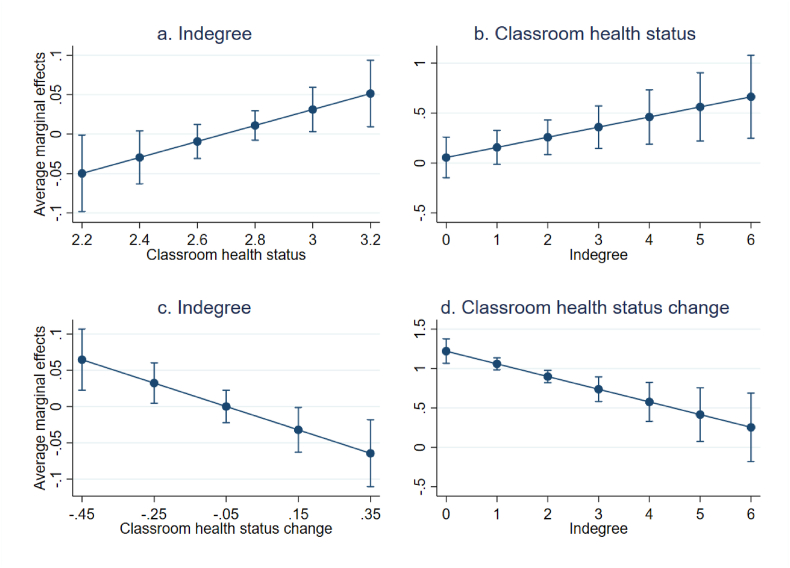
Marginal effects of indegree and classroom health status on adolescents' health status. Note: Figures a. and c. show the marginal effects of indegree on adolescents' health status and health status change across different levels of classroom health status and classroom health status change, respectively. Figures b. and d. show the marginal effects of classroom health status and classroom health status change on adolescents' health status and health status change, respectively.

Models 1b and 2b include the interaction effects between average classroom health and indegree. In model 1b, the interaction term is positive, indegree has a negative effect, and the main effect of classroom health is rendered insignificant. To make this easier to interpret, the marginal effects of classroom health and indegree are plotted in Fig. 1. Fig. 1a shows that in classes with relatively low average health, the effect of indegree on health status is negative, while in class with higher average health, the effect of indegree is positive. When looking at the effect of classroom health on students' health, contingent on indegree (Fig. 1b), the results show that when students’ have low indegree (0 or 1), classroom health is not related to their own health. The higher the number of indegree, the stronger the effect of classroom health.

Model 2b shows the same interaction, but then for change in classroom health over time. The interaction term is negative, the main effect of indegree is nonsignificant, and the effect of change in classroom health becomes somewhat larger compared to the model without the interaction. Fig. 1c shows that when the classroom health deteriorated between the two years, the effect of indegree is positive, while when classroom health improved over time, the effect of indegree is negative. Fig. 1d shows that the higher students’ indegree, the lower the effect of change in classroom health on own health status is. For students with the highest indegree (6) there is even no effect of change in classroom health.

**Fig. 2:**
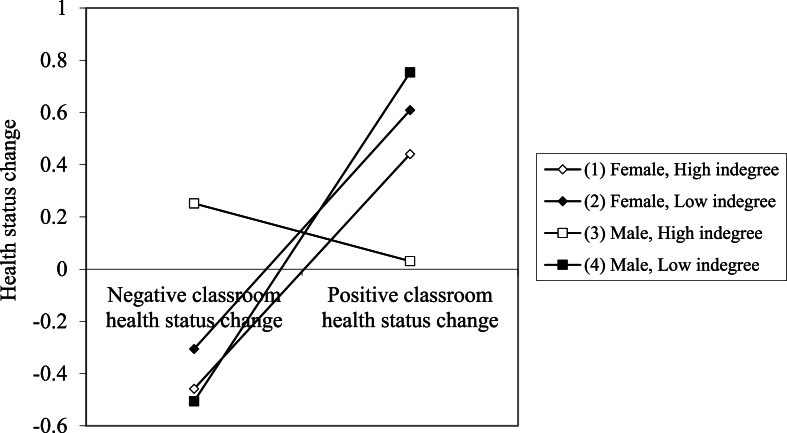
Plot of interaction between indegree, change in classroom health status, and gender. Note: Low and high classroom health change are −.45 and .45, respectively. Low and high indegree are 0 and 6, respectively.

Finally, Fig. 2 shows the plot for the three-way interaction between indegree, change in classroom health status, and gender. The simple slopes for girls with high (b = .999, p = .027) or low indegree (b = 1.016, p < .001) are the same (b_diff = −.017, p = .975), indicating that there is an effect of classroom health, but that there is no interaction between indegree and change in classroom health for girls. The slopes for boys are markedly different; the effect of change in classroom health on own health status for boys with low indegree (b = 1.399, p < .001) is the same as that of girls with low (b_diff = −.383, p = .185) or high indegree (b_diff = −.400, p = .403). However, the effect of classroom health for boys with high indegree is nonsignificant (b = −.245, p = .439), and significantly different from that of boys with low indegree (b_diff = −1.644, p < .001), and girls with low (b_diff = 1.261, p < .001) and high indegree (b_diff = 1.244, p = .041).

## 4 Conclusion and discussion

We studied whether the students' health is related to the health of their classmates, and whether this is contingent on their social position within their class, specifically, their likeability among their classmates. Our findings show that students' own health is indeed linked with that of their classmates: the healthier a students' classmates, the healthier the student. And also, when the average health of classmates increases or decreases over time, students' own health also increases or decreases over that same time, respectively. Thus, students’ health is lined up with that of their classmates.

The existence of direct contextual health effects is, however, not a very novel finding, but we theorized that this contextual effect may not be the same for everyone. Our prediction was that students who are more likeable, are less likely to be influenced by their classroom peers in their health-related behaviors. Our findings tell a seemingly paradoxical story, because the higher students' likeability, the stronger the relation between absolute classroom health and absolute health status, but the weaker the relation between relative classroom health and relative health status. However, the contradiction disappears when we unpack these results. Starting with the cross-sectional analyses on absolute health status, we see that when students are better liked, the effect of classroom health is stronger, but also that the effect of likeability on health is negative in low-health classrooms and positive in high-health classrooms. This is suggestive of a selection effect, where being liked is dependent on behaving in line with the prevalent norms of the classroom. When certain nonhealthy behavior is the norm, this will lead to reduced class health, as well as to a higher likeability of students practicing that behavior. The reverse will be true when healthy behavior is the norm in the class. This finding thus implies reversed causality, in which students’ health status serves as a proxy for health behavior, which gives a signal to classmates who can judge the behavior in light of classroom norms and decide to like the student accordingly.

When we study the results of change in classroom health status between the 7th and 8th grade, we see that the effect of classroom health is strongest for those who are less liked. Better liked students are hardly or not affected by the health in their school class context. Likeability has a positive effect on change in health status in a classroom where average health became worse between the two grades, and a negative effect in a classroom where average health became better between the two grades. These results indicate that likeability serves as a buffer between contextual health and own health. Likeability is an indicator of resilience, where the more resilient someone is, the less likely they will be influenced by their environment, independent of whether that influence is positive or negative. This is in line with the differential susceptibility model, where some individuals are highly malleable by their context, while others are more fixed in their behaviors, regardless of their environment. Because this model examines change in health, it is less likely to be suffering from problems of selection, such as the models examining absolute health. As such, they offer a more compelling argument than the cross-sectional results.

Interestingly, these results were only found for boys, not for girls, suggesting likeability is only a resilience-factor for boys. One explanation for this that boys are generally found to be more susceptible to peer influence in general, including on health-related behavior (Duncan et al., 2005; Erickson et al., 2000). Boys are more susceptible to masculine norms surrounding unhealthy behavior such as alcohol use than girls (Iwamoto & Smiler, 2013), and boys are more likely to be focused on their peer group for friendships, while girls tend to focus on one or a few close friends (Weerman & Hoeve, 2012). Another explanation is that boys are more likely to be exposed to deviant behaviors than girls, because of the composition of their peer group, that is, same-sex peers (Benenson, 1990; Moffitt et al., 2001). If either scenario is true, a measure for resilience is also less likely to be effective. However, there remains a peer effect for girls as well, suggesting that characteristics that were not taken into account may explain girls’ resilience better than likeability. It has been shown that parents intervene differentially in the lives of boys and girls, specifically, parents are more protective of girls, who are more strongly monitored (Kim et al., 1999). For example, it was found that, due to parental strategies, girls were less susceptible to peer influence on alcohol use (Marshal & Chassin, 2000). Missing the variation of parental strategies in the model, may mean that the correct measures for resilience for girls are not in the models.

Opportunities for future work can be found in improving the measurements of health and social networks. We used a broad generalized measure of health or health behavior, resulting in a broad understanding of resilience to peer effects on health. More specific measures can give more detail to this understanding, for example by focusing specifically on smoking, eating behavior, or exercise. This would give insight in exactly what role likeability plays in buffering peer influence on different behaviors, which can contribute to more targeting interventions. Similarly, more detailed measures for networks can improve the understanding of peer effects. The advantage of studying a classroom is that it is quite a stable environment, making the study of change easier. However, students have more people in their networks than just classmates, such as, friends from their neighborhood or sport club. However, because we used Taiwanese data, students in our sample typically spend 10 h per day together at school, leaving them few opportunities to associate with peer outside their class. This implication may be of greater importance when studying a society with a school system where students have more free time.

The results paint a complex picture, in which students who are liked behave according to the class norms when it comes to health-related behaviors, but in which shifts in the class norm do not affect the best liked students. It is possible that high social status is solidified in the first year of secondary school, and cannot easily be lost anymore, while low status students can still gain status throughout the year by conforming to the class's social norms. High likeability serves as a buffer against having to adapt to changing social norms. Regardless, peer influence is a complex phenomenon. Identifying how peer influence works and for whom is crucial to better understand the development of behavior in adolescents. When designing peer-led intervention programs, such as has been done for smoking (e.g., Campbell et al., 2008; Starkey et al., 2009), it is important to understand who are more or less likely to be affected. With intervention programs that hinge on peer relations, it is especially important to understand how different positions within the peer network influence or are influenced, therefore this study adds a crucial piece to that puzzle. The current study contributes to that effort by establishing likeability as a potentially important indicator of resilience that is supportive of individual student health outcomes.

## CRediT authorship contribution statement

**Jaap Nieuwenhuis:** Writing – review & editing, Writing – original draft, Supervision, Methodology, Investigation, Formal analysis, Conceptualization. **Daniël Veennema:** Writing – review & editing, Writing – original draft, Investigation.

## Ethics statement

This study uses secondary data from the Taiwan Youth Project. The authors did not collect data from human subjects.

## Ethics statement

This study uses secondary data from the Taiwan Youth Project. The authors did not collect data from human subjects.

## Funding statement

This study did not receive specific funding.

## Declaration of competing interest

The authors have no competing interests.

## Data Availability

The authors do not have permission to share data.
